# Comparison of volatile/inhalational and IV anesthesia in long-term survival of patients with breast cancer: a retrospective study

**DOI:** 10.1186/s40001-022-00911-9

**Published:** 2022-12-03

**Authors:** Mohammad Yasin Karami, Laleh Dehghanpisheh, Ali Karami, Zahra Sabzloun, Hamid Reza Niazkar, Najmeh Mojarad, Ashkan Panah, Abdolrasoul Talei, Sedigheh Tahmasebi

**Affiliations:** 1 Breast Diseases Research Center, Department of Surgery, Shiraz University of Medical Sciences, Shiraz, Iran; 2 Department of Anesthesiology, Shiraz University of Medical Sciences, Shiraz, Iran; 3 Student Research Committee, Shiraz University of Medical Sciences, Shiraz, Iran

## Abstract

**Objective:**

Breast cancer is a worldwide health concern, and surgical removal has remained the preferred therapeutic option in most patients. Furthermore, the current study was designed to investigate the disease-free survival and overall survival in breast cancer patients, who receive either propofol or isoflurane during operation.

**Method:**

This retrospective study was conducted on 994 patients (IV group, *n* = 530; volatile/inhalational group, *n* = 464) who underwent breast cancer operation from January 2006 to December 2016 at Faghihi Hospital, Shiraz, Iran. All studied patients were followed up till 2020. Patients are classified into two groups, IV and volatile/inhalational, according to the received anesthesia. For statistical analysis, The Cox regression test was conducted to investigate the association between factors affecting the recurrence of the disease and the Log Rank test was utilized to assess the patients’ survival. Finally, to reduce the effect of confounding factors, all patients were matched according to age, tumor size and tumor grade.

**Results:**

Based on results from the log-rank test, the volatile/inhalational group had a better recurrence-free survival (*P* = 0.039) compared to the total IV group. However, the overall survival was not considerably different (*P* = 0.520).

**Conclusion:**

The current study showed that although 2-year disease-free survival is higher in the volatile/inhalational group, there is no meaningful association between the 5-year overall survival and anesthesia technique.

## Introduction

Breast cancer is a global health concern, and it is the most prevalent cancer in women [1, 2]. In addition, breast cancer is the major cause of cancer death in women, responsible for more than five percent of all cancer mortality [1, 3]. Surgical removal of breast cancer remains a mainstay of curative-intent treatment in addition to chemotherapy and radiotherapy [4, 5]. Despite the evident impacts of surgical resection, the operation itself may release the tumor cells into the blood circulation. These tumor cells which are spread during the surgical manipulation may result in metastasis in many patients [3, 6].

Local recurrence or distant metastasis following the initial tumor resection remains a main therapeutic challenge [7]. The likelihood of breast cancer recurrence is multifactorial. Various factors that impact the possibility of metastasis or recurrence, are possible remaining cancer cells of the surgical margin, the type of the cancer cells, and the patient’s immune system [8]. In addition, various predisposing factors such as type of anesthesia, opioid analgesics and physiologic stresses may weaken the immune system and thus accelerate possible metastasis [8, 9]. In this regard, the surgical resection of the tumor may disseminate tumor cells into the vessels [10, 11]. Furthermore, the surgical stress response to tumor removal may trigger the blood cells to release various proinflammatory cytokines, which may impair the immune system, leading to the survival of residual tumor cells, resulting in further metastasis or recurrences [5].

Conventionally, the general anesthesia is established either through administering the volatile inhalational anesthetic which is a halogen-containing hydrocarbon or propofol, which is given intravenously [12]. The present study aimed to assess the overall survival and disease-free survival after surgery in breast cancer patients receiving propofol or isoflurane during the operation.

## Materials and methods

### Study design

The present retrospective cohort study was conducted in Shiraz Breast Clinic, Shiraz, Iran. The mentioned center is the main referral clinic for patients with breast cancers in the South of Iran. In the current study, the clinical data of patients with the impression of Invasive ductal carcinoma who underwent breast cancer surgery were assessed between 2006, and 2016. All studied patients were followed up till 2020. Patients were excluded from the study if met the following criteria: bilateral breast cancer, immediate breast reconstruction surgery, metastatic breast cancer, other malignancy, history of breast surgery, administration of both IV and inhalation anesthetics, male gender, benign breast tumor or carcinoma in situ, American Society of Anesthesiologists [13] physical status greater than or equal to IV, and unknown type of anesthesia or receiving local and regional anesthesia. Patients were categorized based on the received anesthesia into the Total IV (IV group which was treated with continuous infusions of propofol and remifentanil) or volatile/inhalation anesthesia (the volatile/inhalational group which was treated with sevoflurane or isoflurane) and additional analgesic opioids if necessary. The administered anesthesia was selected by the attending anesthesiologists based on the patient’s condition.

### Variables and outcome measurements

The following data were obtained from the recorded data in the breast cancer registry: surgery type, recurrence condition, pre- or post-operation adjuvant chemotherapy, administered radiotherapy or endocrine therapy, invasion condition, tumor size, estrogen and progesterone receptor status, histological tumor grade, histopathological type of tumor, epidermal growth factor receptor type 2 (HER-2) expression, and level of axillary node involvement.

Data regarding the type and the dose of administered analgesics and opioids were gathered through reviewing the hospital records of patients who underwent surgery in Shahid Faghihi hospital affiliated with Shiraz University of medical science. The duration of the operation, duration of anesthesia, and date of surgery were also noted. In this study for reducing the effect of the confounding factors, all the patients were matched according to age, tumor grade, tumor size and hormonal receptor.

### Statistical analysis

The current study results are described as the number and percentage for categorical variables and as the mean ± SD for continuous variables. At first, the normality of the variables was checked through the Kolmogorov–Smirnov test. Considering that the significance value was greater than 0.05. Therefore, all variables have a normal distribution. The independent samples T-test was utilized to assess continuous variables. Also, the Chi-square test was applied in a bid to investigate categorical variables between groups.

In this cohort study, recurrence-free survival and overall survival were calculated for up to 2 and 5 years by conducting the Kaplan–Meier method, and the groups were compared by the log-rank test. The full Cox proportional hazards models were utilized for univariate and multivariate analysis of variables influencing recurrence of breast cancer; potential risk factors such as age, duration of anesthesia, type of anesthesia, involvement of right or left breast, tumor size, tumor grade, in situ component, tumor necrosis, type of lymph node involvement, margin involvement, axillary management, ER, PR, Her2, neoadjuvant and adjuvant chemotherapy, radiotherapy, IORT, and hormone therapy. In addition, backwards multivariable selection were performed. In the backward selection method, all predictive variables are first entered into the equation, and then if they do not meet the criteria to remain in the model, they are removed one by one from the model. Furthermore, the considerable variables (*P* < 0.5) of univariate analysis were considered in multivariate analysis. At the end of this statistical analysis, propensity score matching was applied to decrease the possible confounding impacts of each variable and the variances in baseline characteristics between the groups. The variables used for matching were age, tumor size, and tumor grade. The applied matching method were propensity score matching. It should be noted that these variables were selected based on the previous literature. In this regard, the result of match analysis was like that of the initial analysis. All statistical analyses were conducted with SPSS software version 25.

## Results

Of all patients who went under breast cancer operations from January 2006 to December 2016 at Faghihi Hospital, 994 patients (IV group, *n* = 530; volatile/inhalational group, *n* = 464) were selected for the analyses. The demographic characteristics of studied patients are depicted in Table 1. Breast cancer prognostic factors are shown in Table 2. There were meaningful differences among the propofol group and the sevoflurane group in the duration of follow-up, time to recurrence, axillary management, multi-focal tumor, distribution of estrogen and progesterone receptors, and IORT. The mean follow-up duration was 68.01 (interquartile range, 57.43–78.60) months for the volatile/inhalational group, and 48.83 (interquartile range, 41.36 to 56.30) months for the IV group.

**Table 1:** Demographic characteristics of studied patients

Variables	MEAN ± SD		*P*-value
	Volatile/inhalational	IV	
Age	49.15 ± 12.07	48.40 ± 11.9	0.314
Weight	68.71 ± 11.31	70.53 ± 12.43	0.464
Tumor size	2.79 ± 1.11	2.58 ± 1.13	0.433
Fentanyl (μg) as premed	108.33 ± 24.48	111.22 ± 29.34	0.640
Morphine (mg)	7.38 ± 2.23	7.38 ± 2.32	0.974
Time to last visit (months)	68.01 ± 36.45	48.83 ± 27.62	0.001
Time to recurrence (months)	43.89 ± 30.01	33.53 ± 24.27	0.048

**Table 2:** Patient characteristics for the total study cohort

Variables	Volatile/inhalational (*n* = 464)	IV (*n* = 530)	*P*-value
Breast			0.693
Left	230 (49.5)	268 (50.6)	
Right	230 (49.5)	254 (47.9)	
Missing	4 (0.1)	8 (1.5)	
Axillary management			0.009
ALND	160 (34.5)	182 (33.7)	
SLNB	205 (44.2)	224 (42.3)	
SLNB and ALND	82 (17.7)	109 (20.4)	
Missing	17 (3.6)	15 (3.1)	
Type of tumor			0.412
Invasive ductal carcinoma	458 (98.7)	517 (97.5)	
Other types	6 (1.3)	13 (2.5)	
Tumor size			0.237
(< 2)	103 (22.2)	137 (25.8)	
(2–5)	318 (68.5)	329 (62.2)	
(> 5)	21 (4.5)	24 (4.5)	
Missing	22 (4.8)	40 (7.5)	
Tumor grade			0.864
I	117 (25.3)	123 (23.2)	
II	215 (46.3)	245 (46.2)	
III	100 (21.5)	109 (20.6)	
Missing	32 (6.9)	53 (10.0)	
Nuclear grade			0.063
I	117 (25.3)	123 (23.2)	
II	215 (46.3)	245 (46.2)	
III	100 (21.5)	109 (20.6)	
Missing	32 (6.9)	53 (10.0)	
Multifocal			0.001
No	462 (99.5)	513 (96.8)	
Yes	2 (0.5)	17 (3.2)	
In situ component			0.103
No	178 (38.4)	239 (45.1)	
Yes	286 (61.6)	291 (54.9)	
Tumor necrosis			0.213
No	253 (54.5)	300 (56.6)	
Yes	211 (45.5)	230 (43.4)	
Type of lymph node involvement			0.229
Vascular	122 (26.3)	130 (24.5)	
Pre-neural	39 (8.4)	29 (5.5)	
None	216 (46.5)	245 (46.2)	
Vascular/ pre-neural	49 (10.6)	65 (12.3)	
Missing	38 (8.2)	61 (11.5)	
Margin			0.254
Free	462 (99.5)	526 (99.2)	
Positive	2 (0.5)	4 (0.8)	
ER			0.009
No	84 (18.1)	108 (20.4)	
Yes	366 (78.9)	382 (72.1)	
Missing	14 (3.0)	40 (7.5)	
PR			0.006
No	104 (22.5)	131 (24.7)	
Yes	345 (74.3)	356 (67.2)	
Missing	15 (3.2)	43 (8.1)	
Her2			0.63
No	296 (63.8)	360 (68.0)	
Yes	97 (20.9)	92 (17.3)	
Missing	71 (15.3)	78 (14.7)	
Neoadjuvant chemotherapy			0.392
No	444 (95.7)	503 (94.9)	
Yes	20 (4.3)	27 (5.1)	
Adjuvant chemotherapy			0.401
No	44 (9.5)	43 (8.1)	
Yes	420 (90.5)	487 (91.9)	
Radiotherapy			0.253
No	73 (15.7)	100 (18.9)	
Yes	391 (84.3)	430 (81.1)	
IORT			0.042
No	438 (94.3)	482 (90.9)	
Yes	26 (5.7)	48 (9.1)	
Hormone therapy			0.220
No	108 (23.3)	150 (28.3)	
Yes	357 (76.7)	380 (71.7)	
Recurrence			0.958
No	387 (83.4)	444 (83.8)	
Yes	66 (14.2)	75 (14.1)	
Missing	11 (2.4)	11 (2.1)	
Survival			0.669
Live	409 (88.2)	473 (89.3)	
Dead	41 (8.8)	43 (8.1)	
Missing	14 (3.0)	14 (2.6)	

The data are presented as mean ± SD, median, or number (percentage)

ER, estrogen receptor; HER2, human epidermal growth factor 2; IV, intravenous; PR, progesterone receptor

In addition, the Cox regression analysis was applied in a bid to identify risk factors associated with cancer recurrence. Variables associated with the recurrence of breast cancer are demonstrated in Tables 3 and 4. The meaningful variables in univariate analysis were considered as age, type of anesthesia, tumor size greater than 5 cm, tumor grade III, ALND, ER, PR, neoadjuvant chemotherapy, and hormone therapy. In multivariate analysis of significant variables, tumor size and neoadjuvant chemotherapy remained meaningful risk factors for recurrence after operation.

**Table 3:** Univariable Cox regression analysis for disease recurrence

		Exp(B)	95.0% CI for Exp(B)		*P* value
			Lower	Upper	
Age		0.982	0.967	0.998	0.025
Duration of anesthesia		1.00	1.00	1.00	0.077
Type of anesthesia	Isoflurane	1	–	–	0.043
	Propofol	1.412	1.01	2.01	
Breast	Right	1.051	0.740	1.494	0.781
	Left	1	–	–	–
Tumor size	< 2	1	–	–	–
	2–5	1.160	0.745	1.805	0.511
	> 5	3.135	1.580	6.221	0.001
Tumor grade	I	1	–	–	–
	II	1.412	0.878	2.271	0.154
	III	1.897	1.110	3.241	0.019
In situ component	No	1	–	–	–
	Yes	0.696	0.469	1.032	0.071
Tumor necrosis	No	1	–	–	–
	Yes	1.294	0.882	1.898	0.188
Type of lymph node involvement	Vascular	1.093	0.712	1.676	0.685
	Perineural	1.059	0.524	2.139	0.874
	None	1	–	–	–
	Vascular/ perineural	1.536	0.854	2.762	0.152
Margin	Free	1	–	–	–
	Positive	2.542	0.342	3.67	0.362
Axillary management	SLNB	1	–	–	–
	ALND	2.206	1.463	3.328	< 0.001
	SLNB& ALND	0.984	0.544	1.780	0.958
ER	Negative	1	–	–	–
	Positive	0.470	0.315	0.702	< 0.001
PR	Negative	1	–	–	–
	Positive	0.425	0.291	0.621	< 0.001
Her2	Negative	1	–	–	–
	Positive	1.122	0.707	1.781	0.626
Neoadjuvant chemotherapy	No	1	–	–	–
	Yes	5.830	3.190	10.654	< 0.001
Adjuvant chemotherapy	No	1	–	–	–
	Yes	1.305	0.322	5.290	0.709
Radiotherapy	No	1	–	–	–
	Yes	1.105	0.509	2.398	0.800
IORT	No	1	–	–	–
	Yes	0.824	0.333	2.042	0.676
Hormone therapy	No	1	–	–	–
	Yes	0.494	0.321	0.762	< 0.001

**Table 4:** Multivariable full Cox regression analysis for disease recurrence

		Exp(B)	95.0% CI for Exp(B)		*p*.value
			Lower	Upper	
Age		0.980	0.956	1.005	0.123
Type of anesthesia	Volatile/inhalational	1	–	–	0.073
	IV	0.114	0.011	1.22	
Tumor size	< 2	1	–	–	–
	2–5	1.023	0.552	1.893	0.943
	> 5	2.649	1.014	6.921	0.047
Tumor grade	I	1	–	–	–
	II	1.034	0.543	1.971	0.918
	III	1.020	0.469	2.218	0.961
Axillary management	SLNB	1	–	–	–
	ALND	1.191	0.639	2.218	0.582
	SLNB and ALND	1.048	0.495	2.219	0.902
ER	Negative	1	–	–	–
	Positive	0.831	0.179	3.867	0.814
PR	Negative	1	–	–	–
	Positive	0.765	0.266	2.198	0.619
Neoadjuvant chemotherapy	No	1	–	–	–
	Yes	4.241	1.896	9.490	0.000
Hormone therapy	No	1	–	–	–
	Yes	0.614	0.093	4.043	0.612

**Fig. 1:**
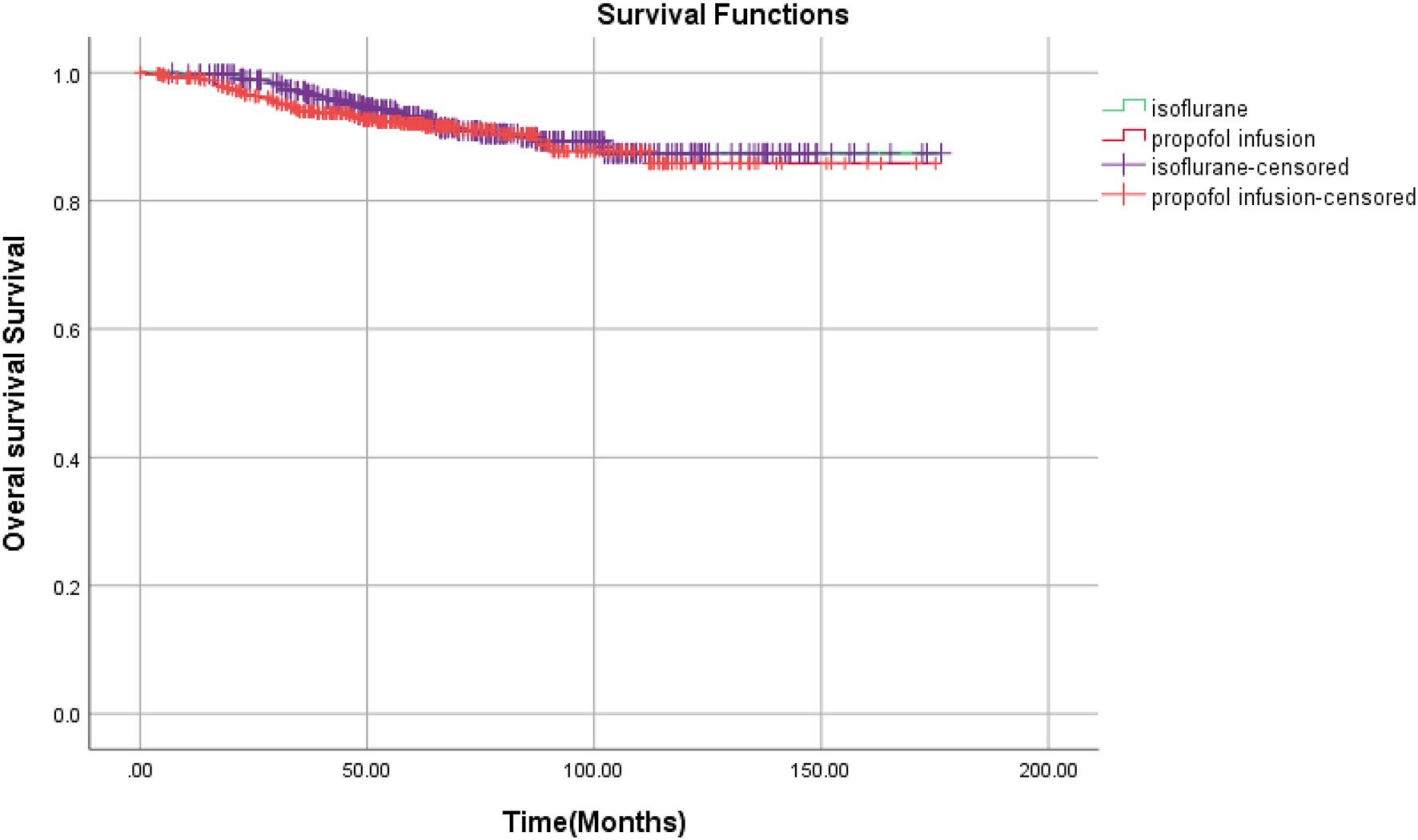
Overall survival

**Fig. 2:**
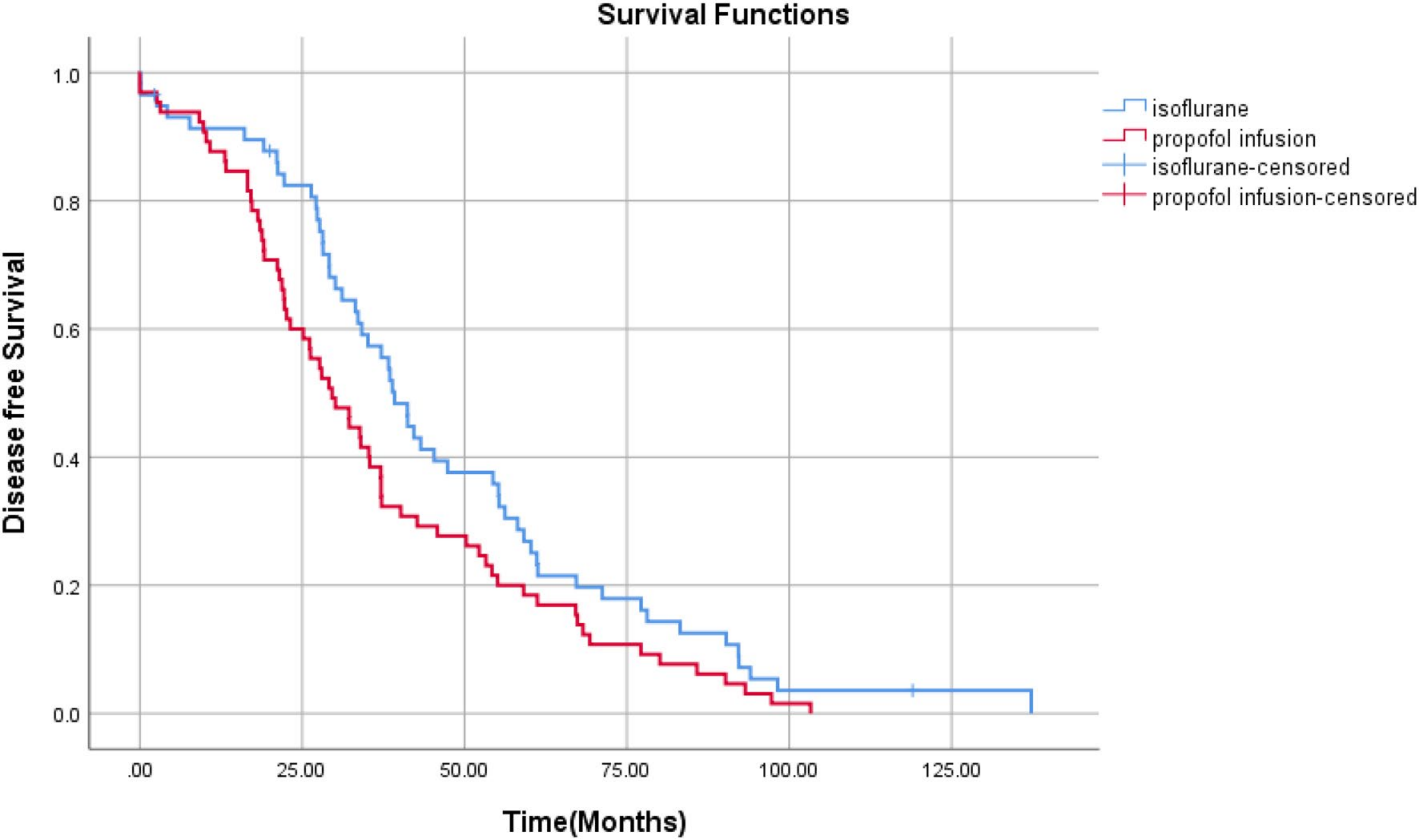
Disease-free survival

Based on results from the log-rank test, the volatile/inhalational group had a better recurrence-free survival (*P* = 0.039) compared to the propofol group. However, the overall survival was not considerably different (*P* = 0.520) (Table 5, Figs. 1 and 2). Table 6 indicates the 2-year recurrence-free survival rates and 5-year overall survival rates for the IV and volatile/inhalational groups. In addition, there is a considerable relationship between IV anesthesia and poorer recurrence-free survival (*P* = 0.015), but there was no considerable difference in 5-year overall survival (*P* = 0.307) between the IV group and the volatile/inhalational group. It should be noted that an Exp (B) greater than 1 indicated an increased risk of recurrence and less than 1 the reverse.

**Table 5:** Disease-free survival and overall survival

	Overall survival			Disease-free survival		
	Mean and 95%CI	Median and 95%CI	*P* value*	Mean and 95%CI	Median and 95%CI	*P* value*
Volatile/inhalational group	161.54 (156.90–166.19)	–	0.520	46.14 (39.13–54.95)	38.5 (34.30–44.14)	0.039
IV group	158.16 (152.85–163.48)	–		36.037 (30.39–42.68)	28.00 (22.85–36.34)	
Overall	160.39 (156.89–163.89)	–		40.71 (36.42–46.41)	34.18 (29.21–39.14)

**Table 6:** 2-year disease-free survival and 5-year overall survival

	Overall survival (5 years)			Disease-free survival (2 years)		
	Proportion terminating	Proportion surviving	*P*-value	Proportion terminating	Proportion surviving	*P*-value
Volatile/inhalational group	7%	93%	0.307	0.18	0.82	0.015
IV group	9%	91%		0.44	0.56	

## Discussion

According to the previous studies, intravenous and inhalational anesthetic drugs may affect the nature of cancer and host immunity not only by triggering cellular receptors and cell signaling pathways, but also by affecting gene transcription [14]. They can also accelerate the progression of cancer metastasis by suppressing several immune systems, such as NK cells, which are known to prevent cancer cell dissemination [6, 8, 15]. Several studies have demonstrated that inhalational anesthetics drugs, including sevoflurane, may accelerate cancer cell spread through up-regulation of hypoxia-inducible factor (HIF) and immunosuppression by harming the natural killer (NK) cell function. In addition, several studies have indicated that propofol may decrease the expression of HIF-1α and prevent tumor growth [3, 16]. Furthermore, Sevoflurane may decrease the function of cell-mediated immunity (CMI) and trigger the apoptosis of T lymphocytes, which leads to increased tumor growth, metastasis and invasion of tumor cells. Contrarily, propofol showed anti-tumor impacts by triggering the differentiation and activation of T helper lymphocytes [17]. Therefore, sevoflurane has a proinflammatory effect and may accelerate the proliferation and metastasis of cancer cells following the breast cancer operation. In addition, anesthetic management with propofol has an anti-inflammatory and antioxidative effect and may decrease the surgery-associated cytokine production and inhibit immunosuppression [3, 18]. In a bid to identify the optimal anesthetics option in patients with breast cancer, the current study was designed to assess the overall survival and disease-free survival after surgery in breast cancer patients receiving propofol or isoflurane during the operation. We demonstrated that patients receiving isoflurane (volatile/inhalational group) had a longer recurrence-free survival (*P* = 0.039) than those receiving propofol (IV group). Nevertheless, the overall survival was not considerably different between these two groups. Currently, there are controversial data considering the optimum anesthesia for patients with cancer. Similar to our findings, Yoo and colleagues [8] in a retrospective study on more than five thousand patients with breast cancer observed no considerable association between the type of anesthesia and recurrence-free or overall survival [8]. In addition, several other studies also reported similar results. However, Wigmore et al. [12] demonstrated that the mortality rate of the inhaled anesthesia patients was statistically higher than those of the intravenous anesthesia group (*P*-value < 0.001, HR = 1.47).

Considering the recurrence-free survival, Yoo et al. [8] observed that disease-free survival was not considerably varied between groups of intravenous anesthesia and inhalation anesthesia, which was inconsistent with our findings. However, in agreement with our study, Kim et al. [19] indicated that in patients with breast cancer, intravenous anesthesia was not associated with disease-free survival compared to inhalation anesthesia (*P*-value = 0.763) [19].

Furthermore, Kyu Oh and colleagues [20] in a retrospective cohort study on 1538 patients with gastric cancer, demonstrated that there is no considerable difference in 1-year survival considering the type of received anesthesia [20]. Nevertheless, Wigmore et al. [12], in a retrospective study on more than seven thousand cancer patients indicated that patients receiving volatile anesthesia had a considerably higher mortality rate during the 3-year follow-up [12]. In addition, another conducted retrospective study on more than one thousand patients with colon cancer observed that propofol-administered patients had better survival in comparison with desflurane-treated patients [21].

Studies indicated that administration of regional anesthesia impacts the immune and inflammatory response of the patient. In this regard, it is assumed that local anesthesia may lead to better outcomes patients with breast cancer [22, 23]. Nevertheless, in the present study patients receiving local anesthesia and paravertebral blocks were excluded.

In addition to the anesthesia method, administration of analgesics may impact the prognosis of breast cancer. Furthermore, a study on 1143 patients with breast cancer indicated that intraoperative administration of opioids improves the recurrence-free survival in patients with triple-negative breast cancer (TNBC) [24].

One of the main strengths of our study is assessing the tumor characteristics such as size, tumor grade, stage, and receptors. In addition, there are several limitations regarding the present study including the small population size, and the single-center, retrospective study design and residual confounding by unmeasured or unknown covariates. Another limitation to our study is the impacts of opium, which is routinely administered pre- and post-operatively in patients with breast cancer. In this regard, in the present study all the patients in both group received opioids for pain relieving. In conclusion, the present study indicated that the overall survival was not considerably different in patients receiving volatile and IV anesthesia. In this regard, further clinical trials are required to identify the optimum anesthesia for patients undergoing cancer surgeries.

## Data Availability

The datasets used and/or analyzed during the current study are available from the corresponding author on reasonable request. We can provide the sources and the data used in the study can be deposited publically.
